# Characterization of Monoclonal Gammopathy of Undetermined Significance by Calorimetric Analysis of Blood Serum Proteome

**DOI:** 10.1371/journal.pone.0120316

**Published:** 2015-03-20

**Authors:** Francisca Barceló, Joan J. Cerdà, Antonio Gutiérrez, Teresa Jimenez-Marco, M. Antonia Durán, Andrés Novo, Teresa Ros, Antonia Sampol, José Portugal

**Affiliations:** 1 Grupo de Investigación Clínica y Translacional, Instituto Universitario de Investigación en Ciencias de la Salud, Universidad de las Islas Baleares, Palma de Mallorca, Spain; 2 Instituto de Física Interdisciplinar y Sistemas Complejos, Universidad de las Islas Baleares-Consejo Superior de Investigaciones Científicas, Palma de Mallorca, Spain; 3 Biología Clínica Hematológica, Hospital Universitario Son Espases, Palma de Mallorca, Spain; 4 Fundación Banco de Sangre y Tejidos de las Islas Baleares, e Instituto Universitario de Investigación en Ciencias de la Salud-Universidad de las Islas Baleares, Palma de Mallorca, Spain; 5 Servicio de Hematología y Hemoterapia, Instituto de Investigación Sanitaria de Palma, Hospital Universitario Son Espases, Palma de Mallorca, Spain; 6 Instituto de Biologia Molecular de Barcelona, Consejo Superior de Investigaciones Científicas, Barcelona, Spain; Universidad de Granada, SPAIN

## Abstract

Monoclonal gammopathy of undetermined significance (MGUS) is a premalignant proliferative disorder that may progress to multiple myeloma, a malignant plasma cell neoplasia. We evaluated differential scanning calorimetry (DSC) as an experimental tool for differentiating serum samples of MGUS patients from healthy individuals. DSC thermograms can be used for monitoring changes in the serum proteome associated with MGUS. MGUS patients showed great variability in serum thermogram characteristics, which depended on the IgG, IgA or IgM isotypes and/or the κ or λ light chains. Thermogram feature parameters distinguished patients with MGUS from healthy people. Serum samples, named as non-MGUS, were also collected from patients with subjacent immunological pathologies who were discarded of having MGUS through serum immunofixation. They were used to verify the sensitivity of DSC for discriminating MGUS from related blood dyscrasias. Only some DSC thermogram feature parameters differentiated, to a lesser extent, between MGUS and non-MGUS individuals. We contemplate DSC as a tool for early diagnosis and monitoring of MGUS.

## Introduction

Monoclonal gammopathy of undetermined significance (MGUS) is a premalignant plasma cell proliferative disorder associated with a life-long risk of progression to multiple myeloma (MM), a malignant neoplasia [1–3]. MGUS etiology remains unclear, yet several studies support a role of both genetic and environmental factors in its development [1,2]. It is the most common plasma cell dyscrasia, prevalent in about 3% of the general population aged 50 years and older [4]. In contrast to the great diversity of normal immunoglobulins, in monoclonal gammopathies a single abnormal cell line predominates, which may produce an intact immunoglobulin, free light chains without heavy chains (often both intact and free), and rarely only heavy chains [1]. Furthermore, each abnormal cell line produces only a κ or λ light chain, but never both of them. Therefore, several distinct clinical subtypes (non-IgM MGUS, IgM MGUS, and light-chain MGUS) have emerged from MGUS as a disease identity [1]. MGUS is defined as having serum M-protein (monoclonal immunoglobulin) < 3 g/dL, clonal plasma cell population in the bone marrow < 10%, and absence of end-organ damage [5,6]. It is unknown whether clinical heterogeneity can ultimately be attributed to simply identifying the original cell of clonal insult or rather the result of a set of complex molecular events that may account for the different clinical subtypes [1]. Little is known about the events that promote the evolution of MGUS and its progression to MM [7,8], yet, based on more than 75,000 individuals, it has been shown that all patients who eventually developed MM were previously diagnosed with MGUS [2].

Several techniques have been traditionally used to detect MGUS. Selection of the preferred technique and correct interpretation of data often depends on an understanding of the immunological basis and pathological conditions associated with MGUS [1,4,9]. Benefit may be expected from analyses using multiparametric immunophenotyping of plasma cells and molecular biology methods, including gene expression analysis [1,10], but it is very difficult to diagnose which MGUS patients will stay stable and those in whom progression to MM will develop [1,5,7]. The status of the M-protein may offer insight into MM development, but this is not absolute, and thus there is a need to identify other biomarkers. The human plasma/serum proteome has to be considered as a suitable specimen for disease diagnosis and therapeutic intervention [11–13]. In clinical practice, it seems interesting to evaluate selected serum molecular biomarkers in MGUS and in the asymptomatic phase of MM.

Differential scanning calorimetry (DSC) can be used to measure the thermal properties of dilute protein solutions as a function of temperature, and it has emerged as a potential method in the analyses of unfractionated blood plasma or serum [13–16]. For a pure protein, DSC provides a unique temperature-induced denaturation profile (thermogram) with a characteristic melting temperature and melting enthalpy. In a protein mixture, such as plasma or serum, the observed thermogram is a composite of the denaturation behavior of the component proteins weighted according to their concentration within the mixture [15,17]. DSC analyses of blood plasma and serum have shown that thermograms obtained from samples of healthy people are highly reproducible with characteristic melting temperatures and well-defined shape [13,16,18]. Serum/plasma of patients suffering from a variety of pathologies showed DSC thermograms that were strikingly different from the thermograms of healthy people [13,16]. Such differences may not be observed by using serum protein electrophoresis [13]. Thermograms obtained from almost any pathological state can be markedly different from one another and this leads to the growing interest in developing calorimetry assays as a clinical diagnostic tool for disease screening. Given that there are grounds for considering that changes in the bulk serum proteome may correlate with the clinical status of certain patients, we sought to substantiate the use of DSC to examine serum from patients with MGUS. DSC thermograms of serum samples distinguished healthy samples from MGUS individuals, and they showed a close connection with different characteristics of MGUS pathology. We contemplate, therefore, DSC as a potential tool for the early diagnosis and monitoring of MGUS.

## Materials and Methods

### Study population and institutional approval

A total of 28 patients (16 men and 12 women) diagnosed with MGUS at the ‘Hospital Universitario Son Espases’ (HUSE) (Palma de Mallorca, Spain), 6 healthy volunteer donors (4 men and 2 women) from the ‘Fundación Banco de Sangre y Tejidos de las Islas Baleares’ (Balearic Islands Blood Bank), Palma de Mallorca, Spain) and 11 non-MGUS individuals (5 men and 6 women)—defined below—from HUSE were recruited for this study. Demographic details are documented in Table 1. The Clinical Research Ethics Committee of the Balearic Islands (CEIC-IB) approved both the study protocol and patient consent procedures (IRB#: IB 1914/12 MB). All the enrolled volunteers gave written informed consent for their blood to be used in this study.

**Table 1:** Patient demographics and disease characteristics.

Sample set^a^	Number of samples	Male/Female	Age range	Age (mean ± SD)
MGUS patients^b^	28	16/12	47–88	71 ± 11
*Healthy* controls	6	4/2	24–66	41 ± 9
Non-MGUS patients^c^	11	5/6	47–84	70 ± 14

^a^ All serum samples were from white people.

^b^MGUS encompasses serum samples of the following isotypes: IgG κ (10 samples), IgG1-κ subclass (2), IgA κ (2), IgM κ (2) IgG λ (7), IgA λ (3), IgM λ (2).

^c^Non-MGUS includes serum samples from patients with subjacent immunological pathologies who were ruled out of having MGUS through serum immunofixation.

The diagnosis of MGUS was based on standard clinical criteria [4] implemented in HUSE. MGUS patients had serum M-protein concentration < 3 g/dL. They were classified according to the monoclonal serum protein as: IgG κ, IgG1-κ subclass, IgG λ, IgA κ, IgA λ, IgM κ, and IgM λ (Table 1). Control groups consisted of both healthy volunteers and a clinical group named as non-MGUS. This non-MGUS group was formed of individuals with subjacent immunological pathologies that were initially suspected of having MGUS—from consideration of some clinical parameters (serum protein electrophoresis among them)—but who were afterwards ruled out of suffering from MGUS by serum immunofixation. All healthy control volunteers were negative in the analytical tests for HIV, Hepatitis B and C, and *Treponema pallidum* infections.

Blood sera coded and stored by the Biobank HUSE or the ‘Fundación Banco de Sangre y Tejidos de las Islas Baleares’ were de-identified before they were delivered to the basic science team. In this way, all serum samples were anonymized and blinded for unbiased data collection. Associated demographic information was collected by the clinical study personnel and provided to the basic science team for data analysis.

### Serum sample collection and preparation

Serum samples of MGUS and non-MGUS patients were obtained at the time of routine clinical procurement. Blood samples from healthy donors were obtained through volunteer donation. Sample collection and handling were conducted according to the approved experimental protocols. Briefly, blood collected in 9 mL red-top glass tubes with serum clot activator (Vacuette España, San Sebastian de los Reyes, Spain) were allowed to stand for 30 min at room temperature and centrifuged at 4.000 rpm in a Heraeus Megafuge (Heraeus SA, Madrid, Spain) for 15 min. Separated serum was transferred into polystyrene tubes (Deltalab, Barcelona, Spain) and stored at 4°C, for less than 48 h before analysis, or at -80°C until use (less than one month). Every serum sample (150 μl) was dialyzed in Slide-A-Lyzer MINI Dialysis Units 2K MWCO (Thermo Scientific, Cultek, Madrid, Spain) for 20 h at 4°C against 100 mL of a solution consisting of 10 mM sodium phosphate, 150 mM NaCl and 15 mM sodium citrate (pH 7.4). The dialyzed samples and the dialysis buffer were filtered using a hydrophobic Millex-HV PVDF 0.45 μm filter (non-sterile 33 mm) (Merck-Millipore, Madrid, Spain). Dialyzed serum samples were 20-fold diluted using filtered dialysis buffer and analyzed immediately.

Total protein concentration in the serum samples was determined colorimetrically in microplates by using the Pierce BCA Protein Assay Kit (Thermo Scientific, Cultek, Madrid, Spain).

### Differential scanning calorimetry (DSC) analysis

DSC measurements were performed using a Nano DSC microcalorimeter (TA Instruments, Cerdanyola del Valles, Spain). Samples were scanned from 30° to 95°C at a scan rate of 1°C/min. Filtered dialysis buffer was used as reference solution. DSC scans were performed in duplicate to ensure the reproducibility of the thermogram profiles for all samples. Data were analyzed using NanoAnalyze software, v. 2.3.6 (TA Instruments). Raw data were corrected for the baseline (buffer *vs*. buffer) and normalized to the total protein concentration. Normalized serum DSC scans were corrected for non-zero baselines by a linear baseline fit. Thermograms were plotted as excess specific heat capacity (cal/°C.g) vs. temperature (°C).

### Analysis of thermogram feature parameters

Several thermogram feature parameters were evaluated for quantitative DSC analysis. We calculated: T_max_, the temperature of the peak maximum, the plot area, Cp1^ex^, the excess specific heat capacity of the first thermal transition, Cp2^ex^, the excess specific heat capacity of the secondary thermal transition, Cp1^ex^/Cp2^ex^, the ratio of the excess specific heat capacities of the first and second transitions, and T_FM_, the first moment temperature (the temperature corresponding to the geometric center of the thermogram), which was calculated as described elsewhere [19]. For every thermogram, all relevant local peaks were analyzed to estimate the temperature maxima of the primary transitions in the 62–65°C range (first thermal transition) and in the 69–75°C range (second thermal transition) and their corresponding excess specific heat capacities, Cp1^ex^ and Cp2^ex^.

Peak deconvolution analysis was performed for the average DSC thermograms of serum samples from healthy individuals. However, that was discarded for MGUS samples and non-MGUS clinical control thermograms because of the wide variability in their DSC profiles (see Results).

### Statistical analysis

Box charts were used for graphical representation of DSC thermogram feature parameters, and to statistically compare differences in the distribution between each study group. Multiple group statistical comparisons of the thermogram feature parameters were assessed by the nonparametric Kruskal-Wallis test. The unpaired Mann-Whitney U test for unequal medians was used for pairwise comparisons between every group. All the statistical analyses were undertaken with the Sage software (v. 6.2) [20], which is freely available at http://www.sagemath.org. Actual p values were calculated, and p < 0.05 was ascribed as statistically significant.

## Results

### Influence of storage temperature and handling of serum samples on DSC thermograms

**Fig 1:**
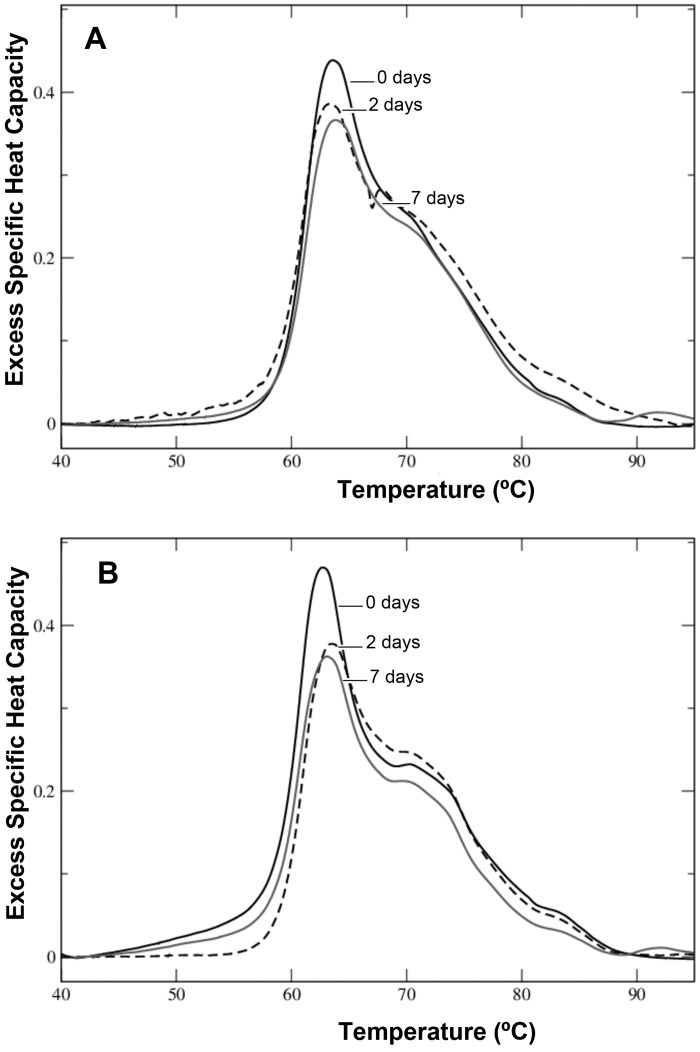
DSC thermograms of blood serum samples stored at 4°C for different periods of time. (A) Thermograms of healthy serum samples analyzed immediately (0 days), and after 2, or 7 days. (B) MGUS-κ serum samples (experimental group based on the κ light chain that is associated with different monoclonal immunoglobulin heavy chains) analyzed after 0, 2, and 7 days.

To perform our studies on MGUS, we gathered sera from patients and healthy individuals obtained at two different institutions: Hospital Universitario Son Espases (HUSE) and ‘Fundación Banco de Sangre y Tejidos de las Islas Baleares’ (Balearic Islands Blood Bank). It was, therefore, our first concern to check whether sample handling and storage conditions, or any delay in delivering them to the DSC facility, may produce differences in the quality of the samples used, as it was to preserve sample integrity that can affect serum protein profiling [21]. To this end, several serum aliquots from healthy or MGUS individuals were either analyzed immediately after their acquisition or stored several days at 4°C before the analysis. No major differences were observed between the thermograms of samples immediately analyzed and those stored at 4°C up to seven days (Fig. 1), but there was a time-dependent decrease in the plot area, and a peak was observed ~ 92°C after seven days storage. Therefore, samples were not stored at 4°C for more than two days before their analysis.

### DSC analysis of healthy controls and MGUS serum samples

**Fig 2:**
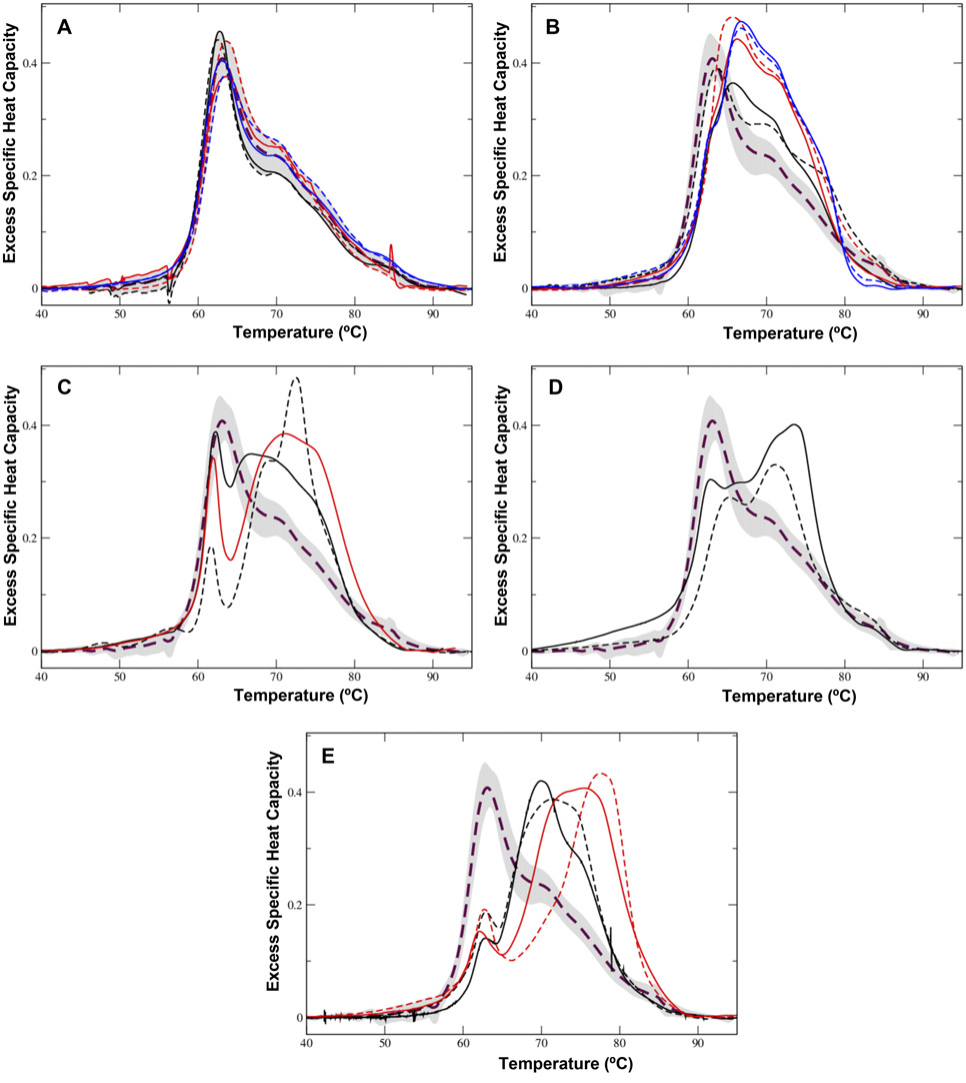
DSC thermograms of serum samples from healthy individuals and patients with MGUS-κ. Samples were gathered as MGUS-κ based on the κ light chain that was found associated with different monoclonal immunoglobulin heavy chains. (A) A set of thermograms obtained from six healthy (control) serum samples. The panel shows an average healthy control plot (dashed brown line) and the standard deviation (shadow). (B) A set of thermograms of serum samples obtained from MGUS patients with IgG κ subtype. (C) Another set of themograms obtained from individuals with IgG κ subtype. For the ease of visualization, thermograms were clustered in either panel B or C based on the similarity of their DSC profiles. (D) Thermograms from patients having IgG1-κ subclass. (E) Thermograms from patients having IgA κ (black) or IgM κ (red) subtypes. For the sake of comparison, the average healthy (control) thermogram (dashed brown line) is presented in all the panels.

Sera from healthy control individuals showed a complex multimodal DSC profile (Fig. 2A) in line with those previously reported for serum and plasma samples [14,16,19,22]. For the current study, control thermograms were averaged and they formed the study healthy control profile. Peak deconvolution analysis of the mean thermogram yielded five peaks, not linked to individual protein components of the serum proteome, with Tm values 62.7°C, 65.8°C, 68.2°C, 70.3°C and 84.1°C, respectively. In the analysis of the healthy thermogram characteristics, we only considered the two peaks that mainly contributed to the cumulative fitted curve (Tm values 62.7°C and 70.3°C, respectively). A (Cp1^ex^/ Cp2^ex^) ratio of 1.55 characterized the average healthy control profile, in keeping with previously reported values [18]. Table 2 shows the thermogram feature parameters of the healthy control group, which includes the median T_max_ value, first moment temperature (T_FM_), and the plot area. Thermograms of sera from individuals having MGUS differed from healthy, control, ones (Fig. 2A-C) except in one case that resembled the healthy DSC profile as implied from both the thermogram shape and the Tm value (cf. Figs. 1B and 2B). Tentatively, this might denote that this serum sample belonged to a patient who was in an “early-stage” of MGUS development.

**Table 2:** Thermogram feature parameters of each clinical group, including “healthy” and non-MGUS sera controls.

Parameter	Healthy^a^	MGUS-kappa^a^	MGUS-lambda^a^	All-MGUS^a^	non-MGUS^a^
Area (cal/g)	5.3 (4.9, 5.5)	6.2 (5.2, 6.5)	6.1 (6.0, 6.4)	6.2 (5.7, 6.5)	5.2 (5.1, 6.1)
T_max_ (°C)	63.3 (62.7, 63.6)	69.9 (65.7, 72.6)	70.5 (69.3, 71.1)	70.3 (66.2, 71.1)	66.6 (65.5, 71.2)
Cp1^ex^ (cal/°C.g)	0.42 (0.38, 0.44)	0.29 (0.19, 0.32)	0.24 (0.18, 0.28)	0.25 (0.18, 0.30)	0.24 (0.19, 0.41)
Cp2^ex^ (cal/°C.g)	0.25 (0.20, 0.26)	0.39 (0.34, 0.42)	0.42 (0.40, 0.45)	0.40 (0.36, 0.43)	0.38 (0.32, 0.39)
Cp1^ex^/Cp2^ex^	1.69 (1.5, 2.1)	0.71 (0.50, 1.00)	0.59 (0.40, 0.70)	0.63 (0.40, 0.80)	0.65 (0.50, 0.90)
T_FM_ (°C)	68.2 (67.6, 68.4)	69.8 (69.1, 70.6)	70.3 (69.9, 71.2)	70.0 (69.2, 70.9)	69.7 (69.0, 70.6)

^a^ Median value (lower and upper value in the first (25th) and third (75th) quartiles).

T_max_, temperature of the peak maximum.

Cp1^ex^, excess specific heat capacity of the first thermal transition.

Cp2^ex^, excess specific heat capacity of the second thermal transition.

Cp1^ex^/Cp2^ex^, ratio of the excess specific heat capacities of the first and second transitions.

T_FM_, first moment temperature.

For an unbiased DSC analysis of diseased sera, MGUS samples were gathered in two major groups based on the light chain associated to the different monoclonal immunoglobulin heavy chains. The resulting groups are named through this paper as MGUS-κ and MGUS-λ, respectively.

Several serum samples from MGUS-κ patients that overexpressed the IgG κ isotype had DSC profiles with a first peak transition in the range 63–67°C and a second transition at ~70–72°C, accompanied by a shoulder at higher temperatures (~77–80°C) (Fig. 2B). Furthermore, some serum samples from patients overexpressing IgG κ (Fig. 2C)—including the IgG1-κ subclass (Fig. 2D)—, IgM κ or IgA κ isotypes (Fig. 2E) showed dissimilar thermograms with clear qualitative differences, which may reflect different stages in the development of MGUS, and the idiosyncrasy of individual serum samples. They showed a defined Tm peak in the 61–63°C range, with large variation in its height value (0.16–0.4 cal/g.deg), together with a main complex peak with Tm values in the 68–77°C range. The DSC thermogram regions around 62–65°C and 69–71°C have been regarded as being dominated by the denaturation of serum albumin and immunoglobulins, respectively [17]. A shoulder around 82–83°C was also observed in some IgG κ, IgA κ and IgM κ thermograms (Fig. 2B-D), which may correspond to the unfolding of transferrin [14] or to a thermally-stabilized transition of other protein because of the serum interactome network. Compared with the average healthy control, the MGUS-κ thermograms were shifted to higher melting temperatures, but, given the large variability observed in the DSC profiles, an average thermogram that could represent the whole diseased MGUS-κ group was not calculated. Nevertheless, DSC thermograms of MGUS-κ serum samples show changes in both the ~62°C and ~70–75°C regions that might be associated with the pathologic status. Table 2 summarizes the quantitative assessments of T_max_ (temperature of the peak maximum), Cp1^ex^ and Cp2^ex^ values at the primary transitions (first and second thermal transitions) and their ratio. The total peak area and the first moment temperature (T_FM_) were also quantified to better describe the thermogram profile (Table 2). The thermogram characteristics quantified for every MGUS-κ sample did not seem to discriminate among IgG κ, IgA κ, and IgM κ isotypes, although this should be treated with caution because of the small experimental sample size which was dictated by the availability of diseased serum samples.

**Fig 3:**
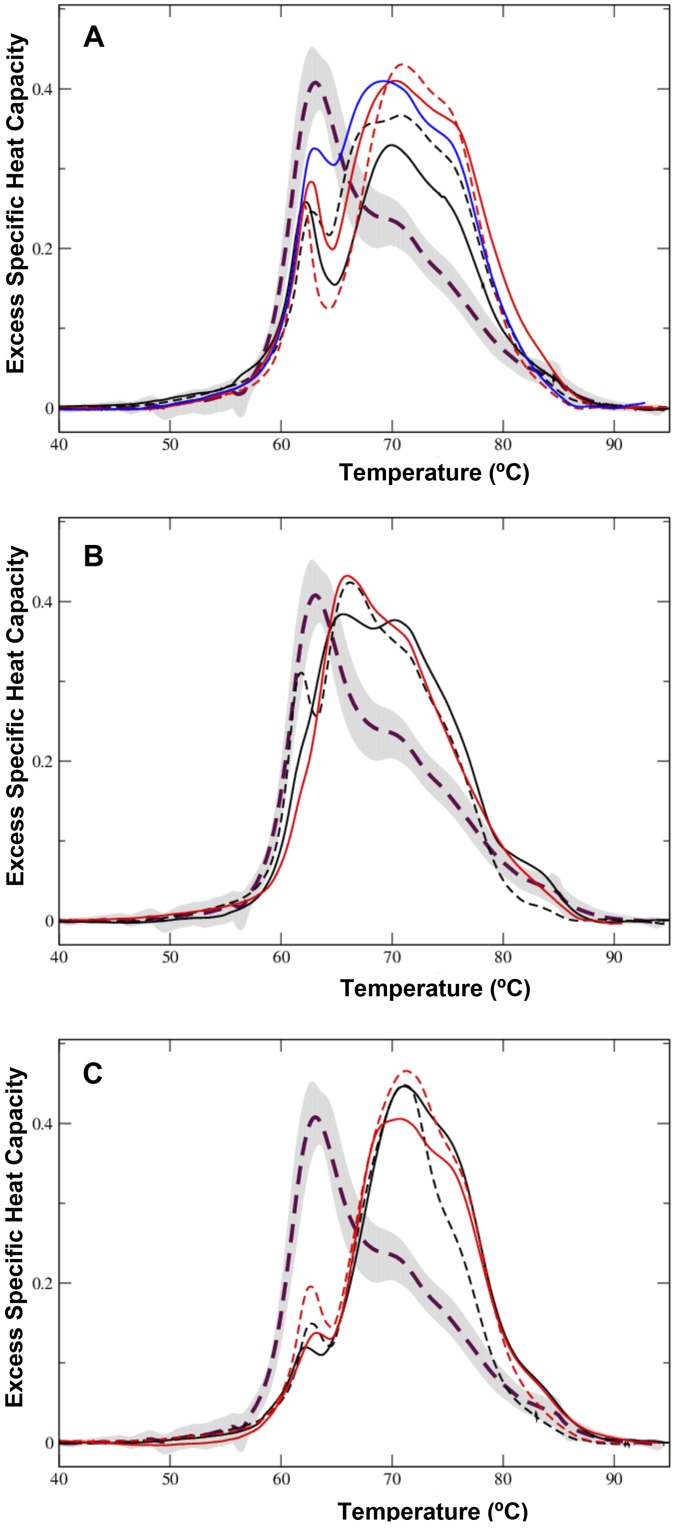
DSC thermograms of serum samples from patients with MGUS-λ. Samples were gathered as MGUS-λ based on the λ light chain that was found associated with the different monoclonal immunoglobulin heavy chains. (A) A set of thermograms of serum samples obtained from patients with IgG λ subtype. (B) A set of thermograms from individuals with MGUS IgG λ subtype (black) or IgM λ subtype (red) grouped together to highlight their similar shape. (C) Several thermograms from patients having IgA λ (continuous black and red plots, and dashed black plot) or IgM λ (dashed red plot) subtypes grouped together because they show similar shapes. For the sake of comparison, the average healthy (control) thermogram is presented in all the panels.

Most DSC thermograms of the MGUS-λ serum samples had a defined peak ~ 62°C with large variance in their height values (0.18–0.28 cal/°C.g), and a second transition (~69–71°C) accompanied by a shoulder in the 75–77°C range (Fig. 3), yet some serum samples of the IgG λ and IgM λ isotypes did not follow such general profile (Fig. 3B). An additional ~ 83°C peak was sometimes observed (Fig. 3), which, as mentioned above, might correspond to the unfolding of transferrin or to a thermally-stabilized transition of other protein owing to the serum interactome network. As for the MGUS-κ group, a large variance in the thermogram parameters was apparent in the MGUS-λ group (Table 2).

### Non-MGUS clinical control samples

**Fig 4:**
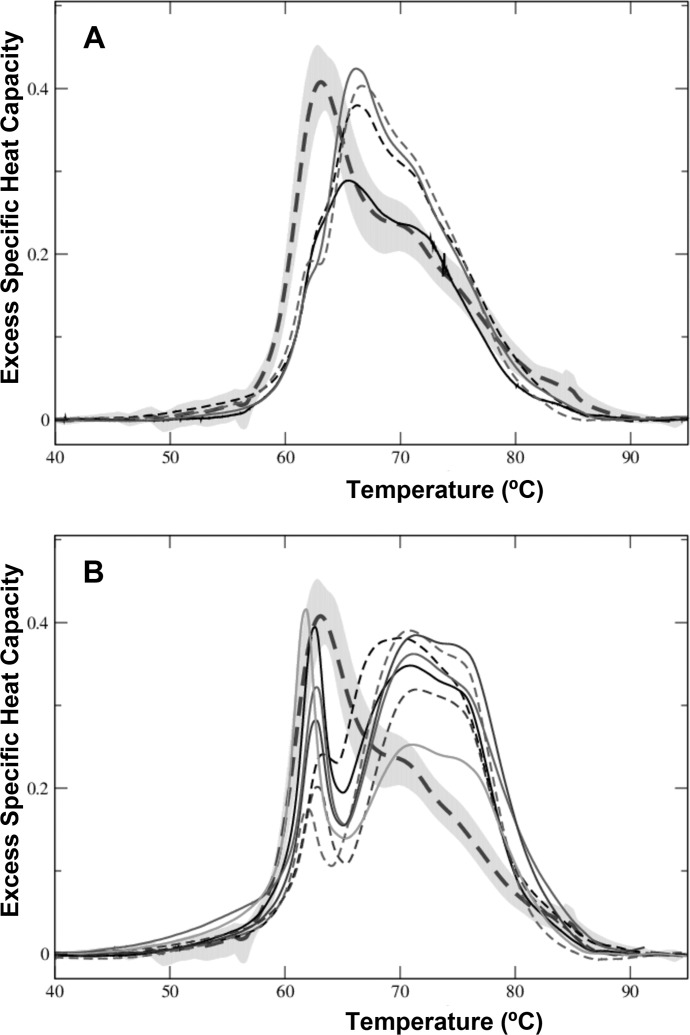
DSC thermograms of serum samples from non-MGUS individuals. Non-MGUS refers to serum samples from individuals with subjacent immunological pathologies who were ruled out of having MGUS through serum immunofixation. (A), (B) Two sets of themograms obtained from non-MGUS individuals. For the ease of visualization, the experimental thermograms were clustered in either panel based on the similarity of the shapes of their DSC profiles. For the sake of comparison, the average healthy (control) thermogram is presented in all the panels.

We also analyzed serum samples from patients with subjacent immunological pathologies, named as non-MGUS, suspected of having MGUS, but ruled out of having such pathology by serum immunofixation. Non-MGUS serum samples were used to examine the sensitivity of DSC to discriminate between MGUS patients and those suffering from related dyscrasias. Non-MGUS samples showed two types of thermogram shapes that were different from those of healthy controls. For the ease of analysis, we grouped the non-MGUS thermograms according to their shape similarities (Fig. 4). A first group displayed a peak transition in the range 65–67°C accompanied by a shoulder at ~70–73°C (Fig. 4A). A second group showed a narrow first transition (61.8–62.8°C) with a large variation in height value (0.19–0.41 cal/°C.g), followed by a complex peak with Tm values in the 68–71°C range (Fig. 4B). Table 2 summarizes the thermogram feature parameters of the non-MGUS serum samples.

### Using DSC thermograms to uncover differences across healthy and diseased groups

**Fig 5:**
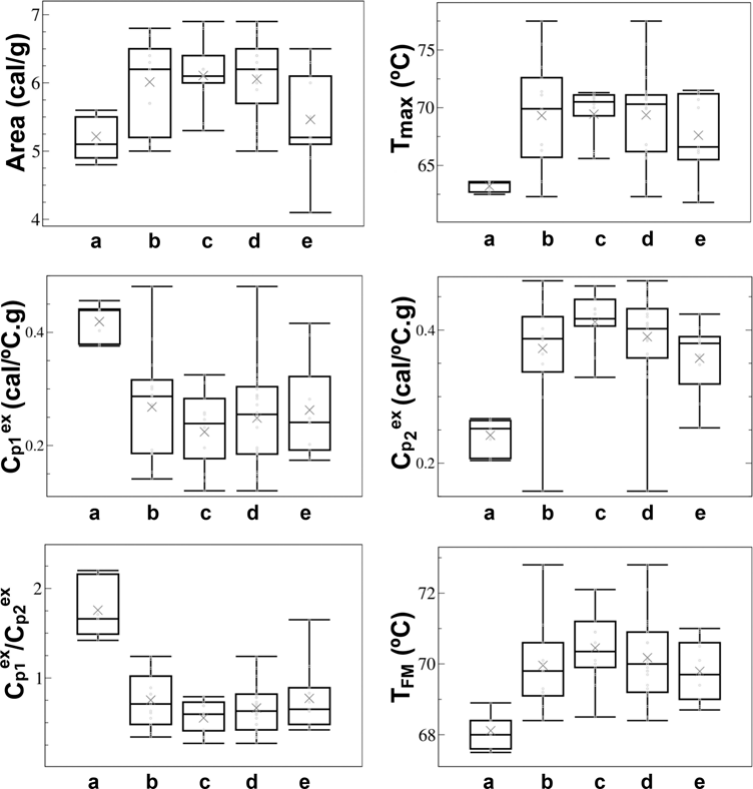
Box chart representations of thermogram feature parameters. For each panel, boxes from left to right represent serum samples from (a) healthy controls, (b) MGUS-κ, (c) MGUS-λ, (d) All-MGUS (which compiles MGUS-κ and MGUS-λ samples together) and (e) non-MGUS. The bottom and top box edges indicate the 25^th^ and 75^th^ percentiles. Within the box, the median value is indicated by the horizontal line, and the mean by the cross inside the box. The whiskers extend from the ends of the boxes to the minimum and maximum experimental values.

Differences in DSC thermograms can indicate a connection between clinical MGUS status and the thermogram characteristics, which might encompass diagnostic possibilities. We sought to gain unbiased insights into whether the thermogram feature parameters described above could be representative indicators of disease-associated changes in the serum proteome. Box plots were used to display variations in MGUS, non-MGUS and healthy serum samples (Fig. 5).

**Fig 6:**
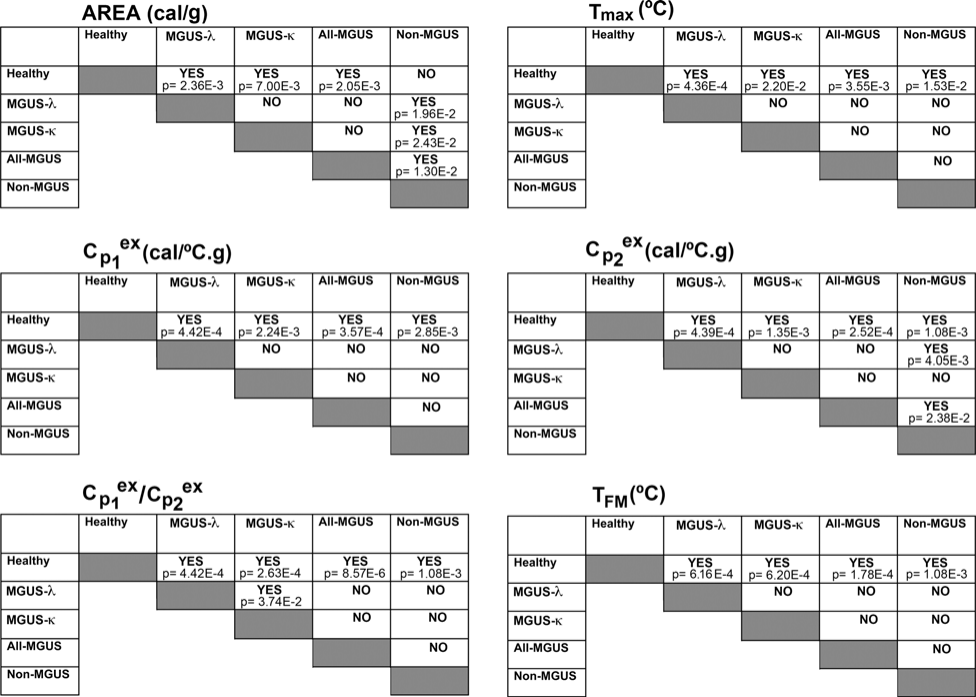
Differences in thermogram feature parameters across the serum sample groups. The thermogram feature parameters are identified at the top of each panel. The analysis of the data by a non-parametric Kruskal-Wallis test revealed significant differences in the six thermogram parameters displayed in the figure (Area, p = 9.82E-3; T_max_, p = 8.73E-3; Cp1^ex^, p = 5.59E-3; Cp2^ex^, p = 4.26E-4; Cp1^ex^/ Cp2^ex^, p = 5.92E-4; T_FM_, p = 2.16E-3), indicating they can be used to distinguish the different serum groups, and also validated the utilization of post-hoc pairwise comparisons. Differences between the serum sample groups were examined for every thermogram parameter by the unpaired Mann-Whitney *U* test. The figure panels indicate “yes” or “no” to specify whether statistically significant differences occurred (the actual p values are shown in the panels).

These plots compare the distribution of six thermogram feature parameters across the diseased and healthy groups. We scrutinized whether the six thermogram characteristics showed significant differences among the different groups of serum data (that is if they showed statistical differences that can be used for distinguishing data for every diseased and healthy groups, as well as among them). In general, trends are observed in the thermogram parameters (Fig. 5) that were statistically corroborated by the non-parametric Kruskal-Wallis one-way analysis of variance. All thermogram feature parameters showed significantly different median values (the actual p values are given in the legend to Fig. 6). This statistical test indicated that significant differences existed among groups, but it did not identify “where” the differences in thermogram characteristics occurred across the different experimental groups. We used, therefore, an unpaired Mann-Whitney U test for unequal medians to evaluate pairwise whether the thermogram parameters can distinguish the different serum groups. Fig. 6 summarizes the comparisons between every set of serum samples and any of the other sets. In this way, we did not only compare healthy serum data with those belonging to MGUS-λ or MGUS-κ groups, but also to non-MGUS set and “all-MGUS” (which compiled λ plus κ sets). The actual p values for every pairwise comparison are itemized inside the panels displayed in Fig. 6. For several thermogram feature parameters, the observed differences were highly significant. Hence, some thermogram features provided us with reliable quantitative measurements of the changes in DSC profiles.

The six thermogram feature parameters differentiated sera of the healthy group from those of the pathological MGUS and non-MGUS groups (see Figs. 5 and 6). The exception was, from a statistical point of view, the DSC thermogram area in the comparison between the healthy and the non-MGUS groups (upper left panel in Fig. 6). The temperature of the peak maximum, T_max_, increased in the MGUS group. The MGUS-κ group showed a wide distribution of T_max_ values that contrast with the narrow range of the MGUS-λ group. Both T_max_ and T_FM_ values increased in the diseased groups when compared to the healthy, control, thermograms (Fig. 5). The first moment temperature (T_FM_) that is a measure of the thermogram shape redistribution, increased in MGUS compared to the healthy control.

The excess heat capacities of the low temperature transitions (Cp1^ex^) were smaller for all the pathological groups than for the healthy one (Fig. 5). This transition would mainly encompass the unfolding of serum albumin [14], and the temperature shift suggests thermal stabilization of that protein in the diseased state by other serum components [17]. Unlike Cp1^ex^, Cp2^ex^ values increased for all diseased groups (Fig. 5). Cp2^ex^ enhancements in people with MGUS can be produced by the thermal stabilization of the unfolding of the pathological monoclonal immunoglobulin (M-protein) through its interaction with other components of the diseased sera, concurring with the denaturation of immunoglobulins described elsewhere [17]. The differences observed between all-MGUS and non-MGUS groups, most significantly in the plot area (Figs. 5 and 6), would reflect that in both blood dyscrasias the immunoglobulins—seemingly the M-protein in MGUS—are actively participating in interactions with other protein and peptide components of the diseased sera in what is defined as the “interactome” [11], which occurred regardless of the exact serum composition that would differentiate these groups.

Comparison between MGUS-κ or MGUS-λ groups and the healthy one revealed highly significant differences in all the thermogram feature parameters (Fig. 6). Therefore, DSC clearly differentiated healthy individuals from those with MGUS. Moreover, the Cp1^ex^/ Cp2^ex^ ratio values were different in the MGUS-κ or MGUS-λ groups (Table 2 and Fig. 5). On the other hand, the thermographs of MGUS-κ, MGUS-λ isotypes and all-MGUS (MGUS-κ plus MGUS-λ groups) differed significantly from the non-MGUS group in the plot area only (Figs. 5 and 6). Notwithstanding the loose definition of the non-MGUS clinical group, it could still be differentiated from MGUS and the healthy serum samples (Fig. 6).

## Discussion

Monoclonal gammopathy of undetermined significance (MGUS) is a premalignant stage that can progress to MM, a malignant plasma cell neoplasia [1,2]. Unfortunately, there are no reliable biological markers that predict which individual with MGUS will progress to MM or related conditions [1,8,23].

Here, we have presented evidences that DSC might be useful as a part of the diagnostic criteria for MGUS. Serum samples from MGUS patients exhibited DSC profiles that differentiate them from those of healthy people (Figs. 2 and 3). Discrimination between healthy and diseased samples has also been described when DSC was used to explore other pathologies [14,16,18,19,24,25]. A general feature of the DSC thermograms of MGUS serum samples was their heterogeneity (Figs. 2–3), which could reflect differences in disease process and progression [3].

Each MGUS subtype follows its own course while displaying individualistic clinical tendencies [1]. The DSC thermograms obtained would correspond to patients diagnosed of having MGUS at diverse stages of disease development. MGUS process can take time before its clinical diagnosed, thus the thermograms of MGUS might reveal the evolution (stages) of the disease. The lack of a single DSC fingerprint for MGUS is consistent with that individual thermograms could indicate different levels of progression of the disease, which it is in line with the abundant differences in the thermogram characteristics described in sera of patients suffering from diverse secretory and non-secretory myeloma types [16,25].

The heterogeneity of DSC thermograms of MGUS disease might reflect disease-associated changes in the serum proteome composition and/or stability of components that affected proteome interactions. The DSC thermogram regions around 62–65°C and 69–71°C have been regarded as being dominated by the denaturation of serum albumin and immunoglobulins, respectively [17]. Our results suggest that both thermogram regions can detect changes in the composition of blood serum that are linked to the MGUS status. The presence of a pathological M-protein, owing to the accumulation of a simple variety of immunoglobulin [1,2,9], would result in changes in the DSC thermogram feature parameters in serum samples from MGUS patients because of altered proteome interactions. Monoclonal immunoglobulin is recognized as a band of restricted migration on serum or urine electrophoresis (M-protein) [9], but, currently, we lack enough clinical data that might allow us to correlate changes in M-protein content and the evolution of MGUS to MM. The relatively small scale of our study precludes us from concluding there is a correlation between the DSC thermograms of patients with MGUS and disease progression, yet the differences observed in the thermogram sets might be related to differences in the disease process and its progression to MM.

Differential diagnosis of MGUS is the determinant for starting therapy [26]. The differences observed in excess of heat capacities among the different MGUS isotypes have a clear diagnostic potential. It will be worth exploring further the Cp1^ex^/ Cp2^ex^ ratio (Fig. 5) as a marker of disease progression. This thermogram feature is highly influenced in MGUS by the interaction of the monoclonal immunoglobulin (M-protein) with other serum protein and peptides, some of which have been described as helpful in MGUS diagnosis [27], even though no single protein is a wholly reliable marker. A non-MGUS clinical control group was included in our DSC studies (Table 1). In this experimental group, serum samples were collected from individuals showing laboratory data compatible with MGUS, but who were ruled out of having it by serum immunofixation. This is a heterogeneous experimental group as deemed by the large variance observed among the DSC thermograms (Fig. 4). Although we have observed differences between non-MGUS and MGUS pathological groups, it is likely that the limited availability of serum samples precludes us from differentiating both diseased groups unambiguously. DSC analysis of serum samples may become a useful diagnostic procedure for MGUS. Our results emphasize that the proteome that could be involved in the transition of MGUS to MM is heterogeneous. DSC thermograms obtained from serum samples reflect clinical differences in protein levels, especially those of the M-protein, and its interactions with other serum biomarkers. It is worth indicating that heterogeneity in MGUS could be linked to ethnic factors [28]. Nevertheless, in our study all samples consisted of blood sera of white people only (Table 1), thus we surmise that ethnical factors had no substantial impact on the heterogeneity of the thermograms shown in Figs. 2–4.

Before DSC could be introduced as a new diagnostic tool, it will be worth examining a larger number of MGUS patients with long-term follow-up to estimate clear correlations between the evolution of the DSC thermograms and disease development. A better understanding of the MGUS pathogenesis and the developing new tools for diagnosis should allow us to define the biological high-risk precursor disease and, ultimately, to develop early clinical intervention strategies.

## References

[1] Korde N, Kristinsson SY, Landgren O (2011) Monoclonal gammopathy of undetermined significance (MGUS) and smoldering multiple myeloma (SMM): novel biological insights and development of early treatment strategies. Blood 117: 5573–5581. 10.1182/blood-2011-01-270140 21441462 PMC3316455

[2] Landgren O, Kyle RA, Pfeiffer RM, Katzmann JA, Caporaso NE, Hayes RB, et al (2009) Monoclonal gammopathy of undetermined significance (MGUS) consistently precedes multiple myeloma: a prospective study. Blood 113: 5412–5417. 10.1182/blood-2008-12-194241 19179464 PMC2689042

[3] Gutiérrez NC, Garcia-Sanz R, San Miguel JF (2007) Molecular biology of myeloma. Clin Transl Oncol 9: 618–624. 17974522 10.1007/s12094-007-0114-4

[4] Kyle RA, Therneau TM, Rajkumar SV, Larson DR, Plevak MF, Offord JR, et al (2006) Prevalence of monoclonal gammopathy of undetermined significance. N Engl J Med 354: 1362–1369. 16571879 10.1056/NEJMoa054494

[5] Kyle RA, Rajkumar SV (2006) Monoclonal gammopathy of undetermined significance. Br J Haematol 134: 573–589. 16938117 10.1111/j.1365-2141.2006.06235.x

[6] Mangiacavalli S, Cocito F, Pochintesta L, Pascutto C, Ferretti V, Varettoni M, et al (2013) Monoclonal gammopathy of undetermined significance: a new proposal of workup. Eur J Haematol 91: 356–360. 10.1111/ejh.12172 23859528

[7] Bladé J, Rosiñol L, Cibeira MT, de Larrea CF (2008) Pathogenesis and progression of monoclonal gammopathy of undetermined significance. Leukemia 22: 1651–1657. 10.1038/leu.2008.203 18668131

[8] Anderson KC, Carrasco RD (2011) Pathogenesis of myeloma. Annu Rev Pathol 6: 249–274. 10.1146/annurev-pathol-011110-130249 21261519

[9] Attaelmannan M, Levinson SS (2000) Understanding and identifying monoclonal gammopathies. Clin Chem 46: 1230–1238. 10926917

[10] López-Corral L, Corchete LA, Sarasquete ME, Mateos MV, Garcia-Sanz R, Fermiñán E, et al (2014) Transcriptome analysis reveals molecular profiles associated with evolving steps of monoclonal gammopathies. Haematologica 99: 1365–1372. 10.3324/haematol.2013.087809 24816239 PMC4116836

[11] Zhou M, Lucas DA, Chan KC, Issaq HJ, Petricoin EF 3rd, Liotta LA, et al (2004) An investigation into the human serum "interactome". Electrophoresis 25: 1289–1298. 15174051 10.1002/elps.200405866

[12] Mor G, Visintin I, Lai Y, Zhao H, Schwartz P, Rutherford T, et al (2005) Serum protein markers for early detection of ovarian cancer. Proc Natl Acad Sci USA 102: 7677–7682. 15890779 10.1073/pnas.0502178102 PMC1140439

[13] Garbett NC, Miller JJ, Jenson AB, Miller DM, Chaires JB (2007) Interrogation of the plasma proteome with differential scanning calorimetry. Clin Chem 53: 2012–2014. 18030697 10.1373/clinchem.2007.091165

[14] Garbett NC, Mekmaysy CS, Helm CW, Jenson AB, Chaires JB, Miller JJ, et al (2009) Differential scanning calorimetry of blood plasma for clinical diagnosis and monitoring. Interrogation of the plasma proteome with differential scanning calorimetry. Exp Mol Pathol 86: 186–191. 10.1016/j.yexmp.2008.12.001 19146849

[15] Michnik A, Drzazga S (2010) Thermal denaturation of mixtures of human serum proteins J Therm Anal Calorim 101: 513–518.

[16] Todinova S, Krumova S, Gartcheva L, Robeerst C, Taneva SG (2011) Microcalorimetry of blood serum proteome: a modified interaction network in the multiple myeloma case. Anal Chem 83: 7992–7998. 10.1021/ac202055m 21928840

[17] Garbett NC, Miller JJ, Jenson AB, Chaires JB (2008) Calorimetry outside the box: a new window into the plasma proteome. Biophys J 94: 1377–1383. 17951300 10.1529/biophysj.107.119453 PMC2212685

[18] Garbett NC, Merchant ML, Chaires JB, Klein JB (2013) Calorimetric analysis of the plasma proteome: Identification of type 1 diabetes patients with early renal function decline. Biochim Biophys Acta 1830: 4675–4680. 10.1016/j.bbagen.2013.05.007 23665587 PMC3743444

[19] Garbett NC, Merchant ML, Helm CW, Jenson AB, Klein JB, Chaires JB (2014) Detection of cervical cancer biomarker patterns in blood plasma and urine by differential scanning calorimetry and mass spectrometry. PLoS One 9: e84710 10.1371/journal.pone.0084710 24416269 PMC3885574

[20] Stein WA (2012) Sage Mathematics Software. (version 6.2) Available: http://sagemath.org

[21] Engwegen JY, Alberts M, Knol JC, Jimenez CR, Depla AC, Tuynman H, et al (2008) Influence of variations in sample handling on SELDI-TOF MS serum protein profiles for colorectal cancer. Proteomics Clin Appl 2: 936–945 10.1002/prca.200780068 21136891

[22] Michnik A, Drzazga Z, Michalik K, Barczyk A, Santura I, Sozańska E, et al (2010) Differential scanning calorimetry study of blood serum in chronic obstructive pulmonary disease. J Therm Anal Calorim 102: 57–60.

[23] Roeker LE, Larson DR, Kyle RA, Kumar S, Dispenzieri A, Rajkumar SV (2013) Risk of acute leukemia and myelodysplastic syndromes in patients with monoclonal gammopathy of undetermined significance (MGUS): a population-based study of 17 315 patients. Leukemia 27: 1391–1393. 10.1038/leu.2013.34 23380709 PMC3676476

[24] Todinova S, Krumova S, Kurtev P, Dimitrov V, Djongov L, Dudunkov Z, et al (2012) Calorimetry-based profiling of blood plasma from colorectal cancer patients. Biochim Biophys Acta 1820: 1879–1885. 10.1016/j.bbagen.2012.08.001 22903026

[25] Todinova S, Krumova S, Radoeva R, Gartcheva L, Taneva SG (2014) Calorimetric markers of Bence Jones and nonsecretory multiple myeloma serum proteome. Anal Chem 86: 12355–12361. 10.1021/ac503677d 25478781

[26] Palladino C, Bruno B, Boccadoro M (2014) Discovering the meaning of monoclonal gammopathy of undetermined significance: current knowledge, future challenges. Transl Med UniSa 8: 12–18 24778994 PMC4000459

[27] Scudla V, Petrova P, Minarik J, Pika T, Bacovsky J (2011) Analysis of the serum levels of selected biological parameters in monoclonal gammopathy of undetermined significance and different stages of multiple myeloma. Neoplasma 58: 499–506 21895403

[28] Landgren O, Graubard BI, Katzmann JA, Kyle RA, Ahmadizadeh I, Clark R, et al (2014) Racial disparities in the prevalence of monoclonal gammopathies: a population-based study of 12 482 persons from the National Health and Nutritional Examination Survey. Leukemia 28: 1537–1542. 10.1038/leu.2014.34 24441287 PMC4090286

